# Zika Virus in Gabon (Central Africa) – 2007: A New Threat from *Aedes albopictus*?

**DOI:** 10.1371/journal.pntd.0002681

**Published:** 2014-02-06

**Authors:** Gilda Grard, Mélanie Caron, Illich Manfred Mombo, Dieudonné Nkoghe, Statiana Mboui Ondo, Davy Jiolle, Didier Fontenille, Christophe Paupy, Eric Maurice Leroy

**Affiliations:** 1 UMVE, Centre International de Recherches Médicales de Franceville, Franceville, Gabon; 2 MIVEGEC, Institut de Recherche pour le Développement (IRD-224, CNRS-5290, Universités de Montpellier 1 & 2), Montpellier, France; 3 Ministère de la Santé Publique, Libreville, Gabon; 4 URES, CIRMF, Franceville, Gabon; Aix Marseille University, France

## Abstract

**Background:**

Chikungunya and dengue viruses emerged in Gabon in 2007, with large outbreaks primarily affecting the capital Libreville and several northern towns. Both viruses subsequently spread to the south-east of the country, with new outbreaks occurring in 2010. The mosquito species *Aedes albopictus*, that was known as a secondary vector for both viruses, recently invaded the country and was the primary vector involved in the Gabonese outbreaks. We conducted a retrospective study of human sera and mosquitoes collected in Gabon from 2007 to 2010, in order to identify other circulating arboviruses.

**Methodology/Principal Findings:**

Sample collections, including 4312 sera from patients presenting with painful febrile disease, and 4665 mosquitoes belonging to 9 species, split into 247 pools (including 137 pools of *Aedes albopictus*), were screened with molecular biology methods. Five human sera and two *Aedes albopictus* pools, all sampled in an urban setting during the 2007 outbreak, were positive for the flavivirus Zika (ZIKV). The ratio of *Aedes albopictus* pools positive for ZIKV was similar to that positive for dengue virus during the concomitant dengue outbreak suggesting similar mosquito infection rates and, presumably, underlying a human ZIKV outbreak. ZIKV sequences from the envelope and NS3 genes were amplified from a human serum sample. Phylogenetic analysis placed the Gabonese ZIKV at a basal position in the African lineage, pointing to ancestral genetic diversification and spread.

**Conclusions/Significance:**

We provide the first direct evidence of human ZIKV infections in Gabon, and its first occurrence in the Asian tiger mosquito, *Aedes albopictus*. These data reveal an unusual natural life cycle for this virus, occurring in an urban environment, and potentially representing a new emerging threat due to this novel association with a highly invasive vector whose geographic range is still expanding across the globe.

## Author Summary

Not previously considered an important human arboviral pathogen, the epidemic capacity of Zika virus (ZIKV, a dengue-related flavivirus) was revealed by the Micronesia outbreak in 2007, which affected about 5000 persons. Widely distributed throughout tropical areas of Asia and Africa, ZIKV is transmitted by a broad range of mosquito species, most of which are sylvatic or rural, *Aedes aegypti*, an anthropophilic and urban species, being considered the main ZIKV epidemic vector. In a context of emerging arbovirus infections (chikungunya (CHIKV) and dengue (DENV)) in Gabon since 2007, we conducted a retrospective study to detect other, related viruses. In samples collected during the concurrent CHIKV/DENV outbreaks that occurred in the capital city in 2007, we detected ZIKV in both humans and mosquitoes, and notably the Asian mosquito *Aedes albopictus* that recently invaded the country and was the main vector responsible for these outbreaks. We found that the Gabonese ZIKV strain belonged to the African lineage, and phylogenetic analysis suggested ancestral diversification and spread rather than recent introduction. These findings, showing for the first time epidemic ZIKV activity in an urban environment in Central Africa and the presence of ZIKV in the invasive mosquito *Aedes albopictus*, raise the possibility of a new emerging threat to human health.

## Introduction

Zika virus (ZIKV) is a mosquito-borne flavivirus phylogenetically related to dengue viruses. Following its first isolation in 1947 from a sentinel monkey placed in the Zika forest in Uganda [1], serological surveys and viral isolations (reviewed in [2]) suggested that ZIKV (i) ranged widely throughout Africa and Asia, and (ii) circulated according to a zoonotic cycle involving non-human primates and a broad spectrum of potential mosquito vector species.

In Africa, ZIKV has been isolated from humans in western and central countries such as Senegal, Nigeria, Central African Republic and Uganda [3]–[7]. Serological surveys (reviewed in [2]) suggested that its geographic range might extend not only to other West and Central African countries (Sierra Leone, Cameroon, Gabon), but also to eastern (Ethiopia, Kenya, Tanzania and Somalia) and northern Africa (Egypt). ZIKV has also been isolated from mosquitoes collected in Senegal, Ivory Coast, Burkina Faso, Central African Republic and Uganda [1], [6], [8], [9]. These mosquitoes mainly belonged to sylvan or rural species of the genus *Aedes*, and more precisely to the *Aedimorphus*, *Diceromyia* and *Stegomyia* subgenera. The virus has also been isolated in West Africa (Burkina Faso, Senegal and Ivory Coast) [6], [9] and Asia [10] from *Aedes aegypti*, a species being considered the main ZIKV epidemic vector outside Africa [11]. Moreover, *Ae. aegypti* was shown experimentally to be an efficient ZIKV vector [12]–[14].

Despite its apparent broad geographic distribution in Africa and Asia, only sporadic cases of human ZIKV infection have been reported. This virus received little attention until its sudden emergence in Yap Island (Micronesia) in 2007, which involved about 5000 persons [15], [16], revealing its epidemic capacity. Patients develop a mild dengue-like syndrome, including fever, headache, rash, arthralgia and conjunctivitis. This clinical similarity with other, more commonly diagnosed arboviral infections such as chikungunya (CHIKV) and dengue (DENV), might delay the diagnosis and/or lead to underestimation of ZIKV infections.

Here, we report the first direct evidence of ZIKV epidemic activity in Central Africa, and its occurrence in an urban environment during concomitant CHIKV/DENV outbreaks in Libreville, the capital of Gabon, in 2007. We also report the first detection of ZIKV in the Asian tiger mosquito, *Ae. albopictus*. These findings, together with the global geographic expansion of this invasive species and its increasing importance as epidemic vector of arboviruses as exemplified by CHIKV adaptation, suggest that the prerequisites for the emergence and global spread of Zika virus may soon be satisfied.

## Materials and Methods

### Study

In 2007 and 2010, Gabon recorded simultaneous outbreaks of CHIKV (genus *Alphavirus*) and DENV (genus *Flavivirus*) infections. The 2007 outbreaks primarily affected Libreville, the capital of Gabon, and subsequently extended northwards to several other towns [17], while the 2010 outbreaks occurred in the south-eastern provinces [18]. To detect other circulating arboviruses, we conducted a retrospective study based on molecular screening of 4312 sera from symptomatic patients presenting to healthcare centers; 24.7% of the samples were obtained during the 2007 outbreaks, 9.7% during the inter-epidemic period, and 65.5% during the 2010 outbreaks (data not shown). We also analyzed a collection of 4665 mosquitoes captured during the same period and split into 247 pools according to the species, date and sampling site (Table 1, see [18] and [19] for the details of the methodology used for mosquito trapping).

**Table 1:** Mosquito collections screened for Zika virus.

	Libreville 2007							Franceville 2010		Total	
Species	Pools	Mos.	Id. (No.)	ZIKV	CHIKV	DENV	Libreville suburb	Pools	Mos.	Pools (%)	Mos.
*Aedes albopictus*	91	2130	T64 (21)	+	+	−	Nzeng-Ayong	46	571	137 (55.4)	2701
			T713 (25)	+	+	−	Alenkiri				
			T707 (25)	−	−	+	Alenkiri				
			T717 (25)	−	−	+	Alenkiri				
			T723 (25)	−	−	+	Alenkiri				
			T724 (6)	−	+	−	Alenkiri				
			T21 (25)	−	+	−	Avorembam				
			T22 (25)	−	+	−	Avorembam				
			T280 (1)	−	+	−	Bel-Air				
*Aedes aegypti* *	40	853						5	28	45 (18.2)	881
*Aedes simpsoni complex*	10	52						5	36	15 (6.1)	88
*Anopheles gambiae* *	8	72								8 (3.2)	72
*Mansonia africana*	6	86								6 (2.4)	86
*Mansonia uniformis* *	4	99								4 (1.6)	99
*Culex quinquefasciatus*	29	690								29 (11.7)	690
*Culex spp.*	1	22								1 (0.4)	22
*Eretmapodites quinquevittatus* *								2	26	2 (0.8)	26
**Total**	**189**	**4004**						**58**	**661**	**247**	**4665**

* Species in which Zika virus has previously been detected.

(%) The percentage of each mosquito species in the collection is indicated in brackets. Mos.: Number of mosquitoes included in a pool. Id. (No.): Mosquito pool positive for ZIKV, CHIKV or DENV, followed by the total number of included mosquitoes in the pool indicated in brackets.

### Ethics statement

The Centre International de Recherches Médicales de Franceville (CIRMF) and the Gabonese Ministry of Health cooperated in the 2007 and 2010 outbreak response and management, that included blood sampling for laboratory diagnostic and epidemiological survey. The study was approved by our Institutional review board (Conseil scientifique du CIRMF).

Symptomatic patients presented to health care centers for medical examination. All patients were informed that blood sampling was required for laboratory diagnosis of suspected acute infections, such as malaria, dengue or chikungunya fever. During the two outbreaks, given the urgency of diagnosis, only oral consent was obtained for blood sampling and was approved by the institutional review board. However during the active surveillance study that was performed between the two outbreaks (described in reference [18]), written consent could be obtained.

### Virus identification and characterization

Primary molecular screening was based on hemi-nested reverse-transcription PCR (hnRT-PCR) with the generic primers PF1S/PF2Rbis/PF3S targeting highly conserved motifs in the flavivirus polymerase (NS5) gene (280-bp) [20]. Yellow fever virus RNA (vaccinal strain 17D) was used as a positive control. A second screening was performed with a ZIKV-specific real-time PCR method using the primers-probe system ZIKV-1086/ZIKV-1162c/ZIKV-1107-FAM [16], also targeting a short sequence (160 bp) of the NS5 gene.

Virus isolation was attempted on the Vero and C6/36 cell lines but was unsuccessful, presumably because of low viral titers (despite two patients presenting only 1 and 4 days after symptom onset), and unsuitable initial storage conditions. To further characterize the Gabonese ZIKV strains, partial envelope (E) (841 bp) and NS3 (772 bp) gene sequences were amplified by conventional nested RT-PCR with specific primers derived from published ZIKV sequences. The primer pairs targeting the E gene were ZIK-ES1 (TGGGGAAAYGGDTGTGGACTYTTTGG)/ZIK-ER1 (CCYCCRACTGATCCRAARTCCCA) and ZIK-ES2 (GGGAGYYTGGTGACATGYGCYAAGTT)/ZIK-ER2 (CCRATGGTGCTRCCACTCCTRTGCCA). The primer pairs for NS3 amplification were ZIK-NS3FS (GGRGTCTTCCACACYATGTGGCACGTYACA)/ZIK-NS3FR (TTCCTGCCTATRCGYCCYCTCCTCTGRGCAGC) and ZIK-X1 (AGAGTGATAGGACTCTATGG)/ZIK-X2 (GTTGGCRCCCATCTCTGARATGTCAGT).

### Phylogenetic analysis

The E and NS3 sequences obtained from one Gabonese patient were concatenated and analyzed using a set of previously published ZIKV sequences. Phylogenetic relationships were reconstructed with the maximum likelihood algorithm implemented in PhyML [21] (available at http://www.atgc-montpellier.fr/phyml/) with best of NNI (Nearest Neighbor Interchange) and SPR (Subtree Pruning and Regrafting) criteria for tree topology searching, and the GTR model of nucleotide substitutions. The Gamma distribution of rate heterogeneity was set to 4 categories, with a proportion of invariable sites and an alpha parameter estimated from the dataset. Branch support was assessed from 100 bootstrap replicates. Tree reconstructions were also performed by Bayesian inference with MrBayes v3.2 [22] under the GTR+I+G model of nucleotide substitutions, and with the distance neighbor-joining method [23] implemented in MEGA5 [24] with confidence levels estimated for 1000 replicates. To test for phylogenetic discrepancies, tree reconstructions were also performed independently from the envelope dataset and the NS3 dataset with PhyML according to the parameters described above.

The resulting trees were visualized with the FigTree software (Available at: http://tree.bio.ed.ac.uk/software/figtree/), and rooted on midpoint for clarity. The Genbank accession numbers for the Gabonese ZIKV strain are KF270886 (envelope) and KF270887 (NS3).

## Results

### Molecular screening

**Figure 1:**
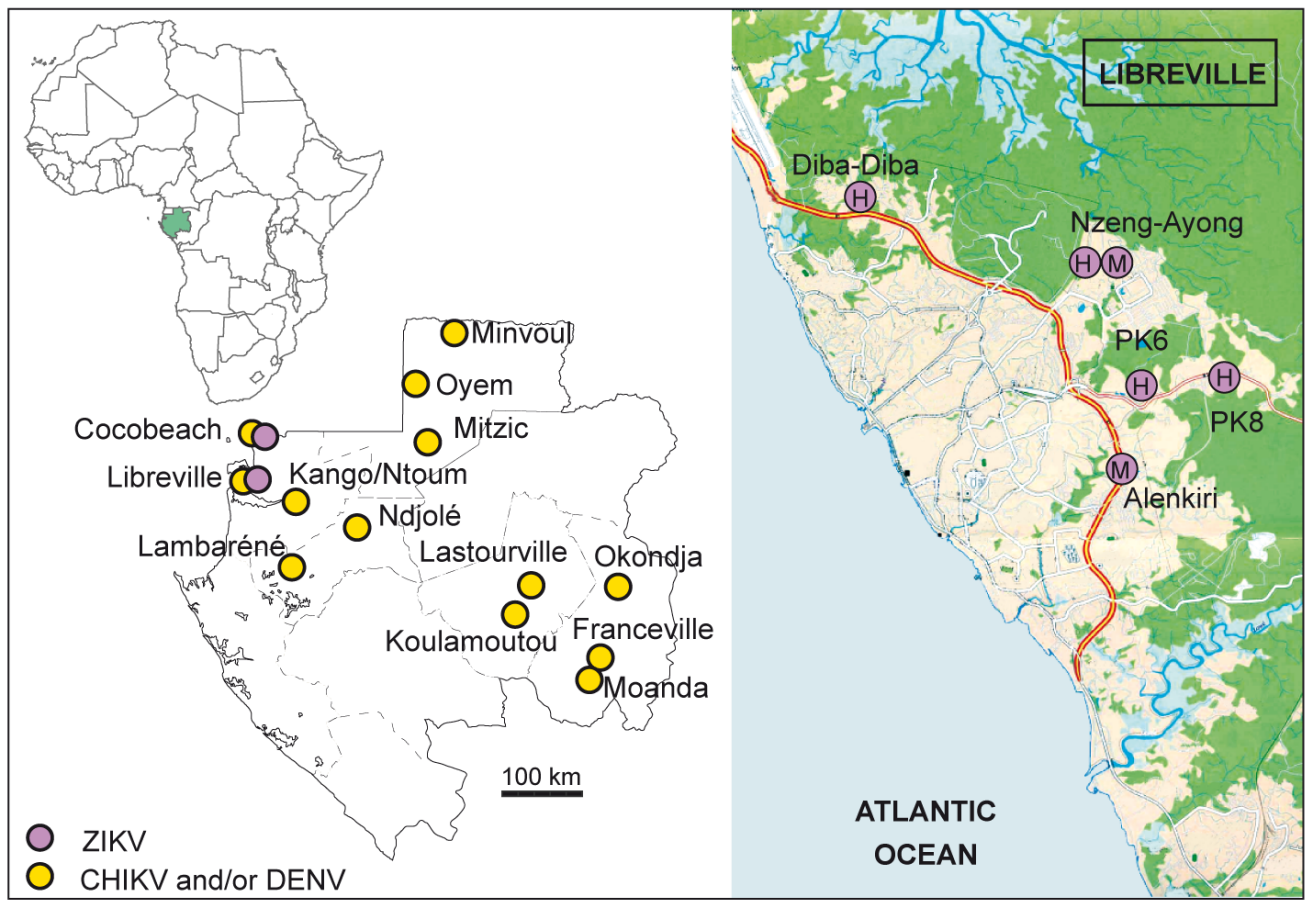
Geographic distribution of Zika and chikungunya and/or dengue viruses infections in Gabon in 2007. The left-hand panel indicates Gabonese CHIKV and/or DENV cases in green circles and ZIKV cases in purple circles. The right-hand panel shows the location of Libreville suburbs where ZIKV-positive human sera (H) and mosquito pools (M) were detected.

The NS5 PCR products were sequenced, resulting in the first ZIKV RNA detection in a human sample (Cocobeach) and in two *Ae. albopictus* pools (Libreville) collected during the 2007 outbreaks. Real-time PCR was then performed, leading to the detection of four additional positive human samples, collected in 2007 in four suburbs of Libreville (Diba-Diba, Nzeng-Ayong, PK8, PK9) (Figure 1). No ZIKV was detected during the inter-epidemic period or during the 2010 outbreaks.

### Clinical description

Clinical information was available for only one ZIKV-positive patient, who had mild arthralgia, subjective fever, headache, rash, mild asthenia, myalgia, diarrhea and vomiting. No information was available on this patient's outcome. Cycle threshold values for human blood samples were high (>37 cycles), suggesting low viral loads (data not shown).

### Vector involvement

*Aedes albopictus* was the predominant species collected, accounting for 55.4% of the mosquito pools, while *Aedes aegypti* accounted for 18.2% (Table 1). The other mosquito species consisted of members of the *Aedes simpsoni complex*, *Anopheles gambiae*, *Mansonia africana*, *Mansonia uniformis*, *Culex quinquefasciatus, Eretmapodites quinquevittatus* and unidentified *Culex* species. Positive mosquito pools were captured from two suburbs (Nzeng-Ayong and Alenkiri) where *Aedes albopictus* was the predominant species (Figure 1, Table 1).

### Sequences analysis

**Figure 2:**
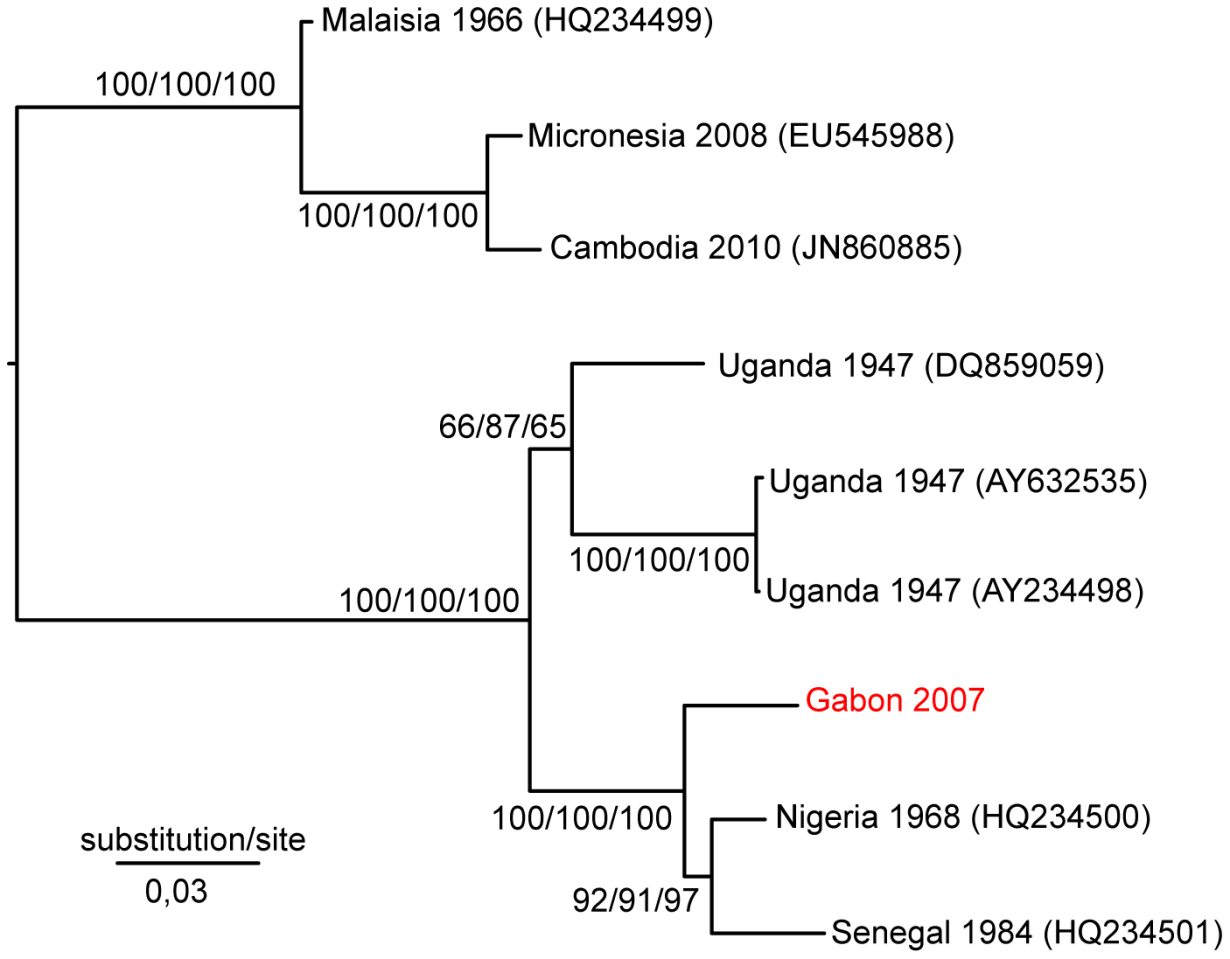
Phylogenetic relationships between concatenated sequences of the Zika virus envelope and NS3 genes. The tree was constructed with the maximum likelihood algorithm implemented in PhyML and rooted on midpoint. Bootstrap values are shown at the respective nodes, followed by bootstrap values resulting from NJ analysis and, finally, the posterior probability resulting from Bayesian analysis. The scale bar indicates the number of substitutions per site. The GenBank accession numbers for the 2007 Gabonese ZIKV isolate are KF270886 (envelope) and KF270887 (NS3).

As isolation on the Vero and C6/36 cell lines failed, the Gabonese ZIKV strain was further characterized by partial sequencing of the E and NS3 genes. Phylogenetic analysis was performed on concatenated E and NS3 sequences from one Cocobeach serum sample. The resulting tree topology (Figure 2) was similar to that previously obtained from the complete coding sequences, corroborating Asian and African distinct lineages [2]. The African lineage was further split into two groups, one containing the genetic variants of the MR766 strain (Uganda, 1947) and the second including West African strains (Nigeria, 1968; Senegal, 1984) and the new ZIKV sequence from Gabon, at a basal position. Phylogenetic trees derived from the E and NS3 partial sequences resulted in a similar topology, apart from the weakly supported branching pattern for the MR766 variant DQ859059, oscillating between the two African sister groups (Supporting Figure S1). The deletions in potential glycosylation sites previously reported for the Nigerian ZIKV strain and two variants of the Ugandan strain MR766 (sequences AY632535 and DQ859059) [2] were absent from the Gabonese ZIKV sequence.

## Discussion

Evidence of human ZIKV infections in Central Africa is limited to one isolate from RCA in 1991 [6] and two serological surveys performed 50 years ago in Gabon [25], [26]. No report of human ZIKV infections was made in other countries of the Congo basin forest block, despite probable circulation through a sylvan natural cycle. We provide here the first direct evidence of human ZIKV infections in Gabon, as well as its occurrence in an urban transmission cycle, and the probable role of *Ae. albopictus* as an epidemic vector.

Our phylogenetic results are in agreement with the tree topology previously obtained with complete coding sequences of ZIKV strains, showing an African lineage and an Asian lineage [2]. The branching pattern obtained here suggests that ZIKV emergence in Gabon did not result from strain importation but rather from the diversification and spread of an ancestral strain belonging to the African lineage. The identification of ZIKV in two different localities of Gabon (Cocobeach and Libreville) suggests that the virus was widespread rather than restricted to a single epidemic focus. The simultaneous occurrence of human and mosquito infections in Libreville also suggests that the virus circulated in 2007 in an epidemic cycle rather than as isolated cases introduced from sylvan cycles.

Of note, ZIKV transmission occurred here in a previously undocumented urban cycle, supporting the potential for urbanization suggested in 2010 by Weaver and Reisen [27]. While some mosquito species (including *Ae. aegypti*) previously found to be associated with ZIKV, were captured and tested here, only *Ae. albopictus* pools were positive for this virus. Moreover, this species largely outnumbered *Ae. aegypti* in the suburbs of Libreville where human cases were detected, suggesting that *Ae. albopictus* played a major role in ZIKV transmission in Libreville.

The ratio of ZIKV-positive *Ae. albopictus* pools is similar to that reported for DENV-positive pools, suggesting that these two viruses infect similar proportions of mosquitoes. The small number of recorded human ZIKV cases, by comparison with DENV cases, may be due to the occurrence of subclinical forms of ZIKV infections that did not required medical attention. Thus, an underlying ZIKV epidemic transmission might have been masked by concomitant CHIKV/DENV outbreaks.

The natural histories of CHIKV and ZIKV display several similarities. Before the large Indian Ocean outbreaks in 2005–2007, chikungunya fever was a neglected arboviral disease. Both viruses are phylogenetically closely related to African viruses [28]–[30] suggesting they probably originated in Africa, where they circulated in an enzootic sylvan cycle involving non-human primates and a wide variety of mosquito species, human outbreaks presumably being mediated by *Ae. aegypti* [5], [31]. In Asia, both viruses are thought to circulate mainly in a human-mosquito cycle involving *Ae. aegypti* [11], [14], [31]. Together with the recent Yap Island outbreak, this prompted some researchers to re-examine the susceptibility of *Ae. aegypti* to ZIKV infection [14]. However, it must be noted that the vector of the Yap Island outbreak was not definitely identified since the predominant potential vector species *Aedes hensilli* remained negative [15], and that ZIKV has been isolated only once from *Ae. aegypti* in Asia [10], so that its vector status *in natura* is not confirmed. Additionally, a ZIKV enzootic transmission cycle involving non-human primates in Asia and sylvatic vectors cannot be ruled out as suggested by serologic studies carried on orangutang [32], [33]. Finally both CHIKV and ZIKV have shown their ability to adapt to a new vector, *Ae. albopictus*, upon introduction in an environment where their primary vector was outnumbered. This mosquito species being native to South-East Asia, our findings may help to explain human ZIKV transmission in Asia.

*Aedes albopictus* was first introduced in Africa in 1991 [34] and detected in Gabon in 2007, where its invasion likely contributed to the emergence of CHIKV and DENV in this country [17]–[19], [35]. Multiple lines of evidence supporting its increasing impact as an arboviral vector have also been obtained during CHIKV outbreaks in the Indian Ocean region (2005–2007) and in Italy (2007) [36], [37] through viral evolutionary convergence of *Ae. albopictus* adaptive mutations [38]–[41]. Whether or not the transmission of ZIKV in Central Africa was also link to such an adaptative mutation of ZIKV to *Ae. albopictus* cannot be answered at this stage. Wong and colleagues [42] have just demonstrated experimentally that *Ae. albopictus* strains from Singapore were orally receptive to the Ugandan strain of ZIKV sampled in 1947, suggesting that this virus-vector association in Africa may have been previously prevented because the required ecological conditions did not yet exist. However, given the relatively low ZIKV viral loads previously reported in patients - with an order of magnitude of 10^5^ copies/ml compared to 10^7^ to 10^9^ copies/ml for CHIKV [16], [18], [40] - the oral infectivity for *Ae. albopictus* may seem at least as critical as it was for CHIKV in establishing this new human-mosquito cycle.

Why ZIKV has not yet been detected in the areas where DENV and CHIKV have already spread *via Ae. albopictus* is unclear, but it may be an ongoing process which we are just starting to detect. The spread of CHIKV reflects the ability of arboviruses to adapt to alternative hosts, and the resulting public health concerns in both developed and developing countries. Is ZIKV the next virus to succeed CHIKV as an emerging global threat? The increasing geographic range of *Ae. albopictus* in Africa, Europe, and the Americas [34], [36], [43], [44], together with the ongoing ZIKV outbreak in French Polynesia at the time of writing [45] suggest this possibility should be seriously considered. Analysis of sylvan and urban transmission cycles, together with viral genetics and vector competence studies, are now required to assess (i) how ZIKV is able to establish a sustainable transmission cycle involving this new vector in Central Africa, (ii) vector(s)-virus relationships in Asia, and (iii) the risk of importation and spread to new areas where *Ae. albopictus* occurs as well.

## Supporting Information

Figure S1 Phylogenetic trees reconstructed from the E and NS3 datasets. Analyses were performed with the maximum likelihood algorythm implemented in PhyML and parameters set as described in the Methods section. Trees are rooted on midpoint.
(TIF)

## References

[1] Dick GW, Kitchen SF, Haddow AJ (1952) Zika virus. I. Isolations and serological specificity. Trans R Soc Trop Med Hyg 46: 509–520. 12995440 10.1016/0035-9203(52)90042-4

[2] Haddow AD, Schuh AJ, Yasuda CY, Kasper MR, Heang V, et al (2012) Genetic characterization of Zika virus strains: geographic expansion of the Asian lineage. PLoS Negl Trop Dis 6: e1477. 22389730 10.1371/journal.pntd.0001477 PMC3289602

[3] Monlun E, Zeller H, Le Guenno B, Traore-Lamizana M, Hervy JP, et al (1993) [Surveillance of the circulation of arbovirus of medical interest in the region of eastern Senegal]. Bull Soc Pathol Exot 86: 21–28. 8099299

[4] Moore DL, Causey OR, Carey DE, Reddy S, Cooke AR, et al (1975) Arthropod-borne viral infections of man in Nigeria, 1964–1970. Ann Trop Med Parasitol 69: 49–64. 1124969 10.1080/00034983.1975.11686983

[5] Fagbami AH (1979) Zika virus infections in Nigeria: virological and seroepidemiological investigations in Oyo State. J Hyg (Lond) 83: 213–219. 489960 10.1017/s0022172400025997 PMC2129900

[6] Adam F, Digoutte JP (2005): Pasteur Institute and IRD. CRORA database. Last access 2013-06-11. Access: http://www.pasteur.fr/recherche/banques/CRORA.

[7] Simpson DI (1964) Zika Virus Infection in Man. Trans R Soc Trop Med Hyg 58: 335–338. 14175744

[8] Haddow AJ, Williams MC, Woodall JP, Simpson DI, Goma LK (1964) Twelve Isolations of Zika Virus from Aedes (Stegomyia) Africanus (Theobald) Taken in and above a Uganda Forest. Bull World Health Organ 31: 57–69. 14230895 PMC2555143

[9] Akoua-Koffi C, Diarrassouba S, Benie VB, Ngbichi JM, Bozoua T, et al (2001) [Investigation surrounding a fatal case of yellow fever in Cote d'Ivoire in 1999]. Bull Soc Pathol Exot 94: 227–230. 11681215

[10] Marchette NJ, Garcia R, Rudnick A (1969) Isolation of Zika virus from Aedes aegypti mosquitoes in Malaysia. Am J Trop Med Hyg 18: 411–415. 4976739 10.4269/ajtmh.1969.18.411

[11] Olson JG, Ksiazek TG, Triwibowo Suhandiman (1981) Zika virus, a cause of fever in Central Java, Indonesia. Trans R Soc Trop Med Hyg 75: 389–393. 6275577 10.1016/0035-9203(81)90100-0

[12] Boorman JP, Porterfield JS (1956) A simple technique for infection of mosquitoes with viruses; transmission of Zika virus. Trans R Soc Trop Med Hyg 50: 238–242. 13337908 10.1016/0035-9203(56)90029-3

[13] Cornet M, Robin Y, Adam C, Valade M, Calvo MA (1979) Transmission expérimentale comparée du virus amaril et du virus Zika chez Aedes aegypti. L Cahiers ORSTOM série Entomologie médicale et Parasitologie. pp. 47–53.

[14] Li MI, Wong PS, Ng LC, Tan CH (2012) Oral susceptibility of Singapore Aedes (Stegomyia) aegypti (Linnaeus) to Zika virus. PLoS Negl Trop Dis 6: e1792. 22953014 10.1371/journal.pntd.0001792 PMC3429392

[15] Duffy MR, Chen TH, Hancock WT, Powers AM, Kool JL, et al (2009) Zika virus outbreak on Yap Island, Federated States of Micronesia. N Engl J Med 360: 2536–2543. 19516034 10.1056/NEJMoa0805715

[16] Lanciotti RS, Kosoy OL, Laven JJ, Velez JO, Lambert AJ, et al (2008) Genetic and serologic properties of Zika virus associated with an epidemic, Yap State, Micronesia, 2007. Emerg Infect Dis 14: 1232–1239. 18680646 10.3201/eid1408.080287 PMC2600394

[17] Leroy EM, Nkoghe D, Ollomo B, Nze-Nkogue C, Becquart P, et al (2009) Concurrent chikungunya and dengue virus infections during simultaneous outbreaks, Gabon, 2007. Emerg Infect Dis 15: 591–593. 19331740 10.3201/eid1504.080664 PMC2671412

[18] Caron M, Paupy C, Grard G, Becquart P, Mombo I, et al (2012) Recent introduction and rapid dissemination of Chikungunya virus and Dengue virus serotype 2 associated with human and mosquito coinfections in Gabon, central Africa. Clin Infect Dis 55: e45–53. 22670036 10.1093/cid/cis530

[19] Paupy C, Ollomo B, Kamgang B, Moutailler S, Rousset D, et al (2010) Comparative role of Aedes albopictus and Aedes aegypti in the emergence of Dengue and Chikungunya in central Africa. Vector Borne Zoonotic Dis 10: 259–266. 19725769 10.1089/vbz.2009.0005

[20] Moureau G, Temmam S, Gonzalez JP, Charrel RN, Grard G, et al (2007) A real-time RT-PCR method for the universal detection and identification of flaviviruses. Vector Borne Zoonotic Dis 7: 467–477. 18020965 10.1089/vbz.2007.0206

[21] Guindon S, Dufayard JF, Lefort V, Anisimova M, Hordijk W, et al (2010) New algorithms and methods to estimate maximum-likelihood phylogenies: assessing the performance of PhyML 3.0. Syst Biol 59: 307–321. 20525638 10.1093/sysbio/syq010

[22] Ronquist F, Teslenko M, van der Mark P, Ayres DL, Darling A, et al (2012) MrBayes 3.2: efficient Bayesian phylogenetic inference and model choice across a large model space. Syst Biol 61: 539–542. 22357727 10.1093/sysbio/sys029 PMC3329765

[23] Saitou N, Nei M (1987) The neighbor-joining method: a new method for reconstructing phylogenetic trees. Mol Biol Evol 4: 406–425. 3447015 10.1093/oxfordjournals.molbev.a040454

[24] Tamura K, Peterson D, Peterson N, Stecher G, Nei M, et al (2011) MEGA5: molecular evolutionary genetics analysis using maximum likelihood, evolutionary distance, and maximum parsimony methods. Mol Biol Evol 28: 2731–2739. 21546353 10.1093/molbev/msr121 PMC3203626

[25] Jan C, Languillat G, Renaudet J, Robin Y (1978) [A serological survey of arboviruses in Gabon]. Bull Soc Pathol Exot Filiales 71: 140–146. 743766

[26] Saluzzo JF, Gonzalez JP, Herve JP, Georges AJ (1981) [Serological survey for the prevalence of certain arboviruses in the human population of the south-east area of Central African Republic (author's transl)]. Bull Soc Pathol Exot Filiales 74: 490–499. 6274526

[27] Weaver SC, Reisen WK (2010) Present and future arboviral threats. Antiviral Res 85: 328–345. 19857523 10.1016/j.antiviral.2009.10.008 PMC2815176

[28] Volk SM, Chen R, Tsetsarkin KA, Adams AP, Garcia TI, et al (2010) Genome-scale phylogenetic analyses of chikungunya virus reveal independent emergences of recent epidemics and various evolutionary rates. J Virol 84: 6497–6504. 20410280 10.1128/JVI.01603-09 PMC2903258

[29] Powers AM, Brault AC, Tesh RB, Weaver SC (2000) Re-emergence of Chikungunya and O'nyong-nyong viruses: evidence for distinct geographical lineages and distant evolutionary relationships. J Gen Virol 81: 471–479. 10644846 10.1099/0022-1317-81-2-471

[30] Grard G, Moureau G, Charrel RN, Holmes EC, Gould EA, et al (2010) Genomics and evolution of Aedes-borne flaviviruses. J Gen Virol 91: 87–94. 19741066 10.1099/vir.0.014506-0

[31] Her Z, Kam YW, Lin RT, Ng LF (2009) Chikungunya: a bending reality. Microbes Infect 11: 1165–1176. 19747979 10.1016/j.micinf.2009.09.004

[32] Kilbourn AM, Karesh WB, Wolfe ND, Bosi EJ, Cook RA, et al (2003) Health evaluation of free-ranging and semi-captive orangutans (Pongo pygmaeus pygmaeus) in Sabah, Malaysia. J Wildl Dis 39: 73–83. 12685070 10.7589/0090-3558-39.1.73

[33] Wolfe ND, Kilbourn AM, Karesh WB, Rahman HA, Bosi EJ, et al (2001) Sylvatic transmission of arboviruses among Bornean orangutans. Am J Trop Med Hyg 64: 310–316. 11463123 10.4269/ajtmh.2001.64.310

[34] Gratz NG (2004) Critical review of the vector status of Aedes albopictus. Med Vet Entomol 18: 215–227. 15347388 10.1111/j.0269-283X.2004.00513.x

[35] Paupy C, Kassa Kassa F, Caron M, Nkoghe D, Leroy EM (2012) A chikungunya outbreak associated with the vector Aedes albopictus in remote villages of Gabon. Vector Borne Zoonotic Dis 12: 167–169. 22141733 10.1089/vbz.2011.0736

[36] Paupy C, Delatte H, Bagny L, Corbel V, Fontenille D (2009) Aedes albopictus, an arbovirus vector: from the darkness to the light. Microbes Infect 11: 1177–1185. 19450706 10.1016/j.micinf.2009.05.005

[37] Bonilauri P, Bellini R, Calzolari M, Angelini R, Venturi L, et al (2008) Chikungunya virus in Aedes albopictus, Italy. Emerg Infect Dis 14: 852–854. 18439383 10.3201/eid1405.071144 PMC2600251

[38] de Lamballerie X, Leroy E, Charrel RN, Ttsetsarkin K, Higgs S, et al (2008) Chikungunya virus adapts to tiger mosquito via evolutionary convergence: a sign of things to come? Virol J 5: 33. 18304328 10.1186/1743-422X-5-33 PMC2266737

[39] Vazeille M, Moutailler S, Coudrier D, Rousseaux C, Khun H, et al (2007) Two Chikungunya isolates from the outbreak of La Reunion (Indian Ocean) exhibit different patterns of infection in the mosquito, Aedes albopictus. PLoS One 2: e1168. 18000540 10.1371/journal.pone.0001168 PMC2064959

[40] Tsetsarkin KA, Vanlandingham DL, McGee CE, Higgs S (2007) A single mutation in chikungunya virus affects vector specificity and epidemic potential. PLoS Pathog 3: e201. 18069894 10.1371/journal.ppat.0030201 PMC2134949

[41] Tsetsarkin KA, Chen R, Sherman MB, Weaver SC (2011) Chikungunya virus: evolution and genetic determinants of emergence. Curr Opin Virol 1: 310–317. 21966353 10.1016/j.coviro.2011.07.004 PMC3182774

[42] Wong PS, Li MZ, Chong CS, Ng LC, Tan CH (2013) Aedes (Stegomyia) albopictus (Skuse): a potential vector of Zika virus in Singapore. PLoS Negl Trop Dis 7: e2348. 23936579 10.1371/journal.pntd.0002348 PMC3731215

[43] Medlock JM, Hansford KM, Schaffner F, Versteirt V, Hendrickx G, et al (2012) A review of the invasive mosquitoes in Europe: ecology, public health risks, and control options. Vector Borne Zoonotic Dis 12: 435–447. 22448724 10.1089/vbz.2011.0814 PMC3366101

[44] Benedict MQ, Levine RS, Hawley WA, Lounibos LP (2007) Spread of the tiger: global risk of invasion by the mosquito Aedes albopictus. Vector Borne Zoonotic Dis 7: 76–85. 17417960 10.1089/vbz.2006.0562 PMC2212601

[45] Promed-Mail. (2013) Subject: PRO/EDR>Zika virus - French Polynesia. Archive Number: 20131106.2041959. Available at http://www.promedmail.org.

